# Calciprotein particle-activated endothelial cells aggravate smooth muscle cell calcification via paracrine signalling

**DOI:** 10.1007/s00018-025-05702-z

**Published:** 2025-04-26

**Authors:** Lian Feenstra, Lara W. Zeper, Brenda van de Langenberg, Eveline J. E. M. Kahlman, Guido de La Roij, Melanie Reijrink, Benoit Bernay, Laurent Chatre, Jeroen Kuipers, Ben N. G. Giepmans, Mirjam F. Mastik, Wierd Kooistra, Monique E. Lodewijk, Malou Zuidscherwoude, Robert A. Pol, the TransplantLines Investigators, Robert A. Pol, Coby Annema, Stephan J. L. Bakker, Stefan P. Berger, Hans Blokzijl, Frank A. J. A. Bodewes, Marieke T. de Boer, Kevin Damman, Martin H. de Borst, Arjan Diepstra, Gerard Dijkstra, Rianne M. Douwes, Caecilia S. E. Doorenbos, Michele F. Eisenga, Michiel E. Erasmus, C. Tji Gan, Antonio W. Gomes Neto, Eelko Hak, Bouke G. Hepkema, Marius C. van den Heuvel, Frank Klont, Tim J. Knobbe, Daan Kremer, Coretta van Leer-Buter, Henri G. D. Leuvenink, Marco van Londen, Willem S. Lexmond, Vincent E. de Meijer, Hubert G. M. Niesters, Gertrude J. Nieuwenhuis-Moeke, L. Joost van Pelt, Robert J. Porte, Adelita V. Ranchor, Jan Stephan F. Sanders, Marion J. Siebelink, Riemer J. H. J. A. Slart, J. Cas Swarte, Daan J. Touw, Charlotte A. te Velde-Keyzer, Erik A. M. Verschuuren, Michel J. Vos, Rinse K. Weersma, Edward. R. Smith, Guido Krenning, Jeroen H. F. de Baaij, Jan-Luuk Hillebrands, Joost G. J. Hoenderop

**Affiliations:** 1 Department of Pathology and Medical Biology (HPC: EA10), University of Groningen, University Medical Center Groningen, Hanzeplein 1, 9713 GZ Groningen, The Netherlands; 2 Department of Medical Biosciences, Radboud University Medical Center, P.O. Box 9101, 6500HB Nijmegen, The Netherlands; 3 Normandie Univ, UNICAEN, US EMerode, Plateform Proteogen, 14000 Caen, France; 4 Université de Caen Normandie, CNRS, Normandie Université, ISTCT UMR6030, GIP CYCERON, 14000 Caen, France; 5 Department of Biomedical Sciences, University Medical Center of Groningen, University of Groningen, Antonius Deusinglaan 1, 9713 AV Groningen, The Netherlands; 6 Department of Surgery, University Medical Center Groningen, University of Groningen, Hanzeplein 1, 9713 GZ Groningen, The Netherlands; 7 Department of Nephrology, Royal Melbourne Hospital, Parkville, VIC Australia; 8 Department of Medicine, University of Melbourne, Parkville, VIC Australia; 9 Present Address: SEHA Kidney Care, Abu Dhabi Health Services (SEHA), Abdu Dhabi, United Arab Emirates; 10 Department of Clinical Pharmacy and Pharmacology, University Medical Center of Groningen, University of Groningen, Hanzeplein 1, 9713 GZ Groningen, The Netherlands

**Keywords:** Calciprotein particles, Chronic kidney disease, Endothelial cell activation, Paracrine signalling, Vascular calcification

## Abstract

**Background:**

Vascular calcification is highly prevalent in Chronic Kidney Disease (CKD) and is associated with markedly increased cardiovascular risk. High serum phosphate in CKD increases calcification propensity via generation of circulating calciprotein particles (CPP2), crystalline nanoaggregates composed of calcium, phosphate, and serum proteins. CPP2 induce vascular calcification in vascular smooth muscle cells (VSMCs) in vitro. In vivo, endothelial cells, rather than VSMCs are primarily exposed to CPP2, yet understanding the influence of endothelial cells on vascular calcification is limited.

**Methods:**

We investigated calcification-promoting signalling by endothelial cells on VSMCs. Effects of CPP2 exposure to endothelial cells on CPP2 uptake, endothelial cell activation, and endothelial cell-derived secretome were studied. Effects of the secretome on VSMC calcification were investigated. Using NanoString nCounter analysis the effects of CPP2-activated endothelial cell-conditioned medium on VSMCs gene expression were mapped.

**Results:**

Endothelial cells internalise CPP2 and elevate ICAM-1, E-selectin, and VCAM-1-mRNA expression, indicating endothelial activation. VSMCs cultured in conditioned medium from CPP2-activated endothelial cells demonstrated enhanced calcification, suggesting that CPP2-activated endothelial cells release pro-calcifying soluble factors. Mass spectrometry was utilized to identify 1171 proteins in the CPP2-activated endothelial cells’ secretome. Among these, 76 proteins were differentially expressed compared to control endothelial cells’ secretome, including proteins related to blood vessel development, extracellular matrix remodelling, and oxidative stress-related processes. Finally, endothelial cell-derived paracrine factors present in conditioned medium enhanced mRNA-expression of calcification-related factors in VSMCs.

**Conclusions:**

CPP2-activated endothelial cells promote VSMC calcification via paracrine signalling. In response to these paracrine factors, VSMCs increase the expression of pro-calcification genes.

**Supplementary Information:**

The online version contains supplementary material available at 10.1007/s00018-025-05702-z.

## Introduction

Vascular calcification (VC) is associated with cardiovascular morbidity and mortality in chronic kidney disease (CKD) [1, 2]. VC occurs in two forms; intimal and medial calcification, depending on the layer in the vessel wall where calcification occurs. While both forms of VC can be present in CKD, medial calcification (also known as Mönckeberg's sclerosis) is more common in CKD patients [3]. The medial layer of the vasculature consists of vascular smooth muscle cells (VSMCs), and therefore most factors involved in medial VC are studied for their effect on VSMCs. Uremic toxins, hyperphosphatemia, hypercalcemia, hyperparathyroidism, inflammation and oxidative stress are all known factors to be involved in VSMC calcification in CKD [4]. The effects of high serum phosphate levels on VC have been extensively studied as serum phosphate levels are associated with all-cause and cardiovascular mortality in CKD patients [5–7]. High phosphate levels participate in the VC process via the formation of calciprotein particles (CPP), which contain, amongst others, phosphate, calcium and serum proteins. CPP can be present in two forms: small and round primary CPP (CPP1), which spontaneously mature over time into more crystalline and larger secondary CPP (CPP2), which have been suggested to drive the development of VC [8]. In vitro, CPP2 are found to induce VSMC calcification directly [9, 10]. Additionally, CPP2 have been associated with aortic stiffness in predialysis patients as well as increased risk of worse cardiovascular outcomes in patients on dialysis [11, 12].

Endothelial cells (ECs) form a monolayer that lines the lumen of the blood vessels and serves as the cellular barrier between blood and the VSMCs. ECs are known to regulate VSMC function, and endothelial function is declined in patients with CKD [13–15]. Endothelial dysfunction has been associated with the development of vascular pathology. [16] Clinical studies demonstrated that endothelial dysfunction is associated with higher rates of hospitalization, cardiovascular events and all-cause mortality in patients with CKD [17–19]. Interestingly, ECs develop into a more osteoblastic phenotype following exposure to traditional risk factors of CKD such as hyperphosphatemia and inflammation, resulting in endothelial to mesenchymal transition (EndMT) and apoptosis of the ECs. [20, 21] Moreover, CPP2 have been shown to induce mRNA expression of activation markers and upregulate inflammatory markers in ECs [22–24]. Although we previously showed that CPP2 have direct effects on ECs [25], and EC dysfunction is an important cause of CV mortality in CKD[13, 14], the direct contribution of ECs to the development of CPP2-induced VC remains uncharacterized.

A particular mechanism by which ECs could contribute to CPP2-induced VC is by the loss of EC barrier function, which will increase passive CPP2 transfer to the subendothelial space and allows direct exposure of medial VSMCs to CPP2. Previous in vitro studies have shown that direct exposure of VSMCs to CPP2 leads to calcium deposition, TNF-α secretion and osteochondrogenic dedifferentiation of VSMCs [9, 10]. On the other hand, considering the direct effects of CPP2 on ECs in vitro, data advocate for a more active contribution of ECs to the development of CPP2-induced VC. Upon high phosphate exposure, ECs change the cargo of released extracellular vesicles (EVs) resulting in more pro-calcifying vesicles, thereby enhancing VSMC calcification [26–28]. In addition, in response to phosphate and indoxyl sulphate or to CPP2, ECs are known to secrete IL-8, which is a promoter of VSMC calcification by inhibiting the expression of the anti-calcification factor osteopontin [22, 29]. These data suggest the existence of a paracrine interaction between ECs and VSMCs. Nevertheless, data on the paracrine crosstalk between ECs and VSMCs in CPP2-induced VC are lacking, and therefore studies on the effects of CPP2 on ECs and its resulting effect on VSMC calcification are warranted.

Here, we aim to investigate the effect of CPP2-modified EC-mediated paracrine signaling on VSMC calcification. To this end, first the association between EC activation and CPP2 abundance was established in a cohort of CKD patients to provide a rationale for further in-depth mechanistic studies. Next, mass spectrometry and gene set enrichment analysis was performed to define the CPP2-modified ECs secretome in vitro. Effects of EC-derived compounds on VSMC calcification were analyzed in an in vitro EC-VSMC conditioned medium model. Using NanoString nCounter expression analysis, the effect of CPP2-activated ECs conditioned medium on VSMC gene expression was mapped.

## Results

### CPP counts correlate with circulating VCAM-1 levels in vivo

**Fig. 1:**
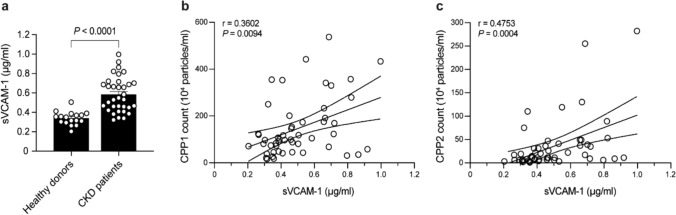
Correlations of soluble VCAM-1 (sVCAM-1) and CPP counts in healthy kidney donor and CKD patients. **a** sVCAM-1 levels (pg/ml) measured in plasma of healthy kidney donors (n = 17) and CKD patients (n = 34). Data presented as mean ± SEM and tested with Mann–Whitney U test. **b** Correlation of sVCAM-1 levels and serum CPP1 counts (10^4^ particles/ml). **c** Correlation of sVCAM-1 levels and serum CPP2 counts (10^4^ particles/ml) (two-tailed nonparametric Spearman correlation). The correlation coefficient is indicated with the symbol r. *P* < 0.05 was considered statistically significant

Previously it was shown that synthetically generated CPP2 activate ECs in vitro.[22, 23] Additionally, serum from CKD patients upregulated expression of EC activation markers in vitro.[24] In order to assess the association between circulating CPP counts and EC activation in humans in vivo, plasma and serum of both healthy kidney donors (N = 17) and CKD patients (N = 34) was collected. Baseline characteristics are summarized in Table 1. Plasma levels of EC activation and dysfunction marker soluble VCAM-1 (sVCAM-1) were measured. Plasma sVCAM-1 levels were significantly increased (1.7-fold,* P* < 0.0001) in CKD patients compared to the healthy donors (Fig. 1a). We recently showed that CKD patients have increased CPP1 and CPP2 counts when compared to healthy living donors. [30] In the combined population of healthy kidney donors and CKD patients serum levels of both CPP1 and CPP2 significantly correlated with plasma sVCAM-1 levels (r = 0.3602, *P* = 0.0094 for CPP1; r = 0.4753, *P* = 0.0004 for CPP2) (Fig. 1b and 1c).

**Table 1:** Baseline characteristics of the study populations: chronic kidney disease (CKD) patients and healthy living kidney donors (KD)

	Patients with chronic kidney disease (CKD, N = 34)	Healthy kidney donors (KD, N = 17)	*P*-value
Sex assigned at birth (% male)	53	59	0.77
Age (years)	55 ± 13	49 ± 12	0.10
Body mass index (kg/m^2^)	27 ± 4.1	26 ± 2.3	0.30
Dialysis (%)	29	0	< 0.001
Estimated glomerular filtration rate (mL/min/1.73m^2^)	8.0 [6.0–11]	92 [75–104]	< 0.001
Type 2 diabetes mellitus (%)	29	0	0.02
Diabetes duration (years)	16 [10–21]*		
HbA1_C_ (%)	5.4 [5.0–6.0]	5.4 [5.2–5.6]	0.77
Total cholesterol (mmol/L)	4.7 ± 1.4	4.8 ± 1.0	0.67
C-reactive protein (mg/L)	2.2 [1.0–6.0]	1.6 [0.6–3.3]	0.18
Leukocyte count (10^9^/L)	7.1 ± 2.1	6.8 ± 2.5^**^	0.63
Calcium (mmol/L)	2.3 ± 0.2	2.4 ± 0.1	0.79
Phosphate (mmol/L)	1.5 [1.3–1.9]	0.91 [0.84–0.95]	< 0.001

^*^N = 10, ^**^N = 16. Data are presented as mean ± SD (when normally distributed) or as median with [IQR] (when not normally distributed). p-value based on unpaired t-test when normally distributed or Mann–Whitney U test when not-normally distributed or Chi-squared test for categorical data (gender, dialysis and type 2 diabetes mellitus)

### ECs become activated and take-up CPP2 from the environment in vitro

Since CPP counts are associated with EC activation and dysfunction in clinical samples in vivo, the interaction between CPP2 and ECs was further explored in different EC in vitro models. Both HUVECs and HAECs showed upregulation of the mRNA expression of EC activation markers ICAM-1, E-selectin and VCAM-1 after exposure to 25 CPP2 for 24 h (Supplementary Figure S1(**_LIFE-D-24-02586_R1**)). The mRNA expression of ICAM-1 and E-selectin was ~ fourfold increased compared to the control conditions (HUVECs ICAM-1, *P* < 0.0001; E-selectin, *P* < 0.01, and HAECs ICAM-1 and E-selectin both *P* < 0.01) (Supplementary Fig. S1a, b and d, e(**_LIFE-D-24-02586_R1**)). The most prominent change in mRNA expression was observed with VCAM-1, showing a ninefold increase in the CPP2-activated ECs compared to the unstimulated ECs (HUVECs *P* < 0.0001; HAECs *P* < 0.01)(Supplementary Fig. S1c, f(**_LIFE-D-24-02586_R1**)).

**Fig. 2:**
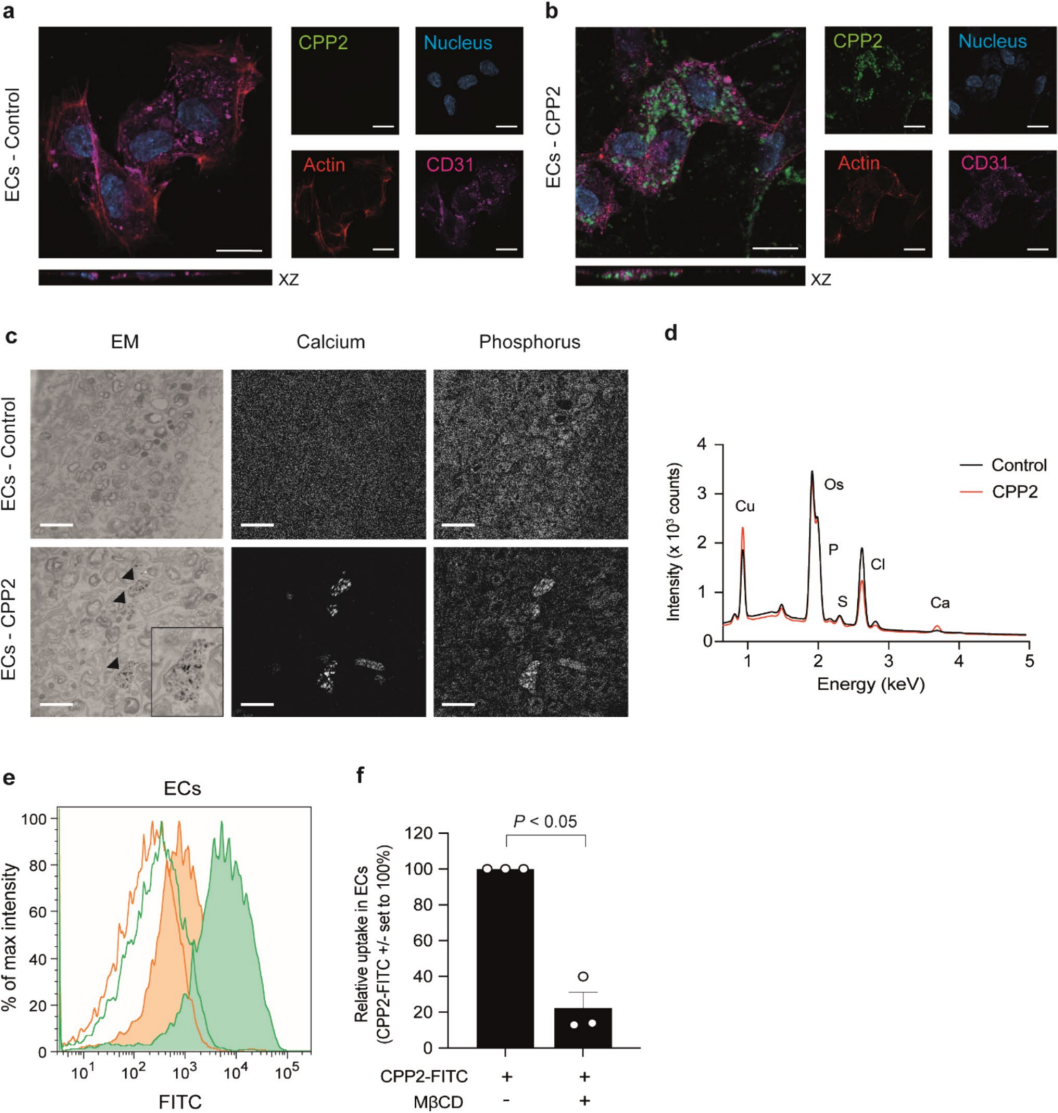
Endothelial uptake op CPP2 in vitro. **a**, **b** Confocal images of ECs treated without (**a**) or with (**b**) FITC-labelled CPP2 (green), 25 µg calcium per ml CPP2 solution (= 25 µg/ml). XZ planes demonstrate internalisation of CPP2. Nucleus = blue, actin skeleton = red and CD31 = pink. Scale bars represent 20 μm. **c** Electron microscopical analysis of ECs showing CPP2 uptake. CPP2-containing vesicles are marked with an arrowhead. Insert in left bottom panel shows a magnification of a CPP2-containing vesicle. Calcium and phosphate content of the particles is visualised with EDX. Scale bars represent 2 μm. **d** EDX spectra showing copper (Cu), osmium (Os), phosphate (P), sulphur (S), chloride (Cl) and calcium (Ca) for unstimulated ECs (control, black line) and the CPP2 stimulated ECs (red line) in ECs. **e** Flow cytometric analysis demonstrated the uptake of FITC-labelled CPP2 (green) and the inhibition thereof in the presence of 2.5 mM methyl-β-cyclodextrin (MβCD; orange). Lines indicate incubation at 4 °C (extracellular binding), filled histograms indicate incubation at 37 °C (endocytosis + extracellular binding). **f** Quantification of CPP2 endocytosis in the absence or presence of endocytosis inhibitor MβCD. Data is presented as mean ± SEM of n = 3 individual experiments (each consisting of 3 biological replicates). One sample t-test was performed against a hypothetical value of 100% and *P* < 0.05 was considered statistically significant

Next, we analyzed the fate of CPP2 once the particles are presented to ECs (HUVECs) in culture. Hereto, CPP2 were fluorescently labelled prior to incubation with ECs. Using confocal microscopy labelled CPP2 were detected intracellularly is ECs (Fig. 2a, b). Additionally, electron microscopy (EM) was performed combined with energy-dispersive x-ray spectroscopy (EDX) to define subcellular localization of internalized CPP2 in the cytoplasm. CPP2-activated ECs showed presence of CPP2-resembling particles in cytoplasmic vesicles (marked with arrowheads, Fig. 2c). EDX spectra of particle-containing vesicles in both CPP2-exposed and control ECs (Fig. 2d) indicated the presence of calcium in the vesicles of CPP2-exposed cells only, an observation consistent with the presence of CPP2 in cytoplasmic vesicles. To quantify the uptake of CPP2, ECs were incubated with FITC-labelled CPP2 and flow cytometric analysis was performed (Fig. 2e and Supplementary Figure S2c(**_LIFE-D-24-02586_R1**)). ECs take-up CPP2 from the environment which could be inhibited by incubating cells at hypometabolic (4 °C) conditions (Fig. 2e, line histograms). To confirm active endocytosis, methyl-β-cyclodextrin (MβCD) was used as an endocytosis inhibitor [31]. MβCD reduced CPP2 uptake by 80% in ECs (Fig. 2e, f, *P* < 0.05). Inhibitor cytochalasin D resulted in similar results as it reduced endocytosis with 91% in ECs (Supplementary Fig. S2f, *P* < 0.001(**_LIFE-D-24-02586_R1**)).

### Absence of classical VSMC dedifferentiation markers and uptake of CPP2 by VSMCs in vitro

To compare responses of ECs and VSMCs towards CPP2 exposure in vitro, also VSMCs were exposed to 25 CPP2 for 24 h in vitro. First, the calcification potential of CPP2 was assessed. CPP2 exposure to VSMCs increased calcium deposition (340 ± 30 µg calcium/mg protein) compared to the non-exposed VSMCs (*P* < 0.05, Supplementary Fig. S3a, b(**_LIFE-D-24-02586_R1**)). Osteogenic markers runt-related transcription factor 2 (RUNX2) and alkaline phosphatase (ALPL) showed a reduction in gene expression of 26% (*P* < 0.0001) and 17% (*P* < 0.001) in CPP2-exposed VSMCs, respectively (Supplementary Fig. S3c, d(**_LIFE-D-24-02586_R1**)). Gene expression of bone morphogenetic protein 2 (BMP2) was almost doubled (1.8-fold increase) in CPP2-exposed versus non-exposed VSMCs (*P* < 0.0001, Supplementary Fig. S3e(**_LIFE-D-24-02586_R1**)). Expression levels of SM22⍺ and MGP were not affected by CPP2 exposure (Supplementary Fig. S3f, g(**_LIFE-D-24-02586_R1**)).

**Fig. 3:**
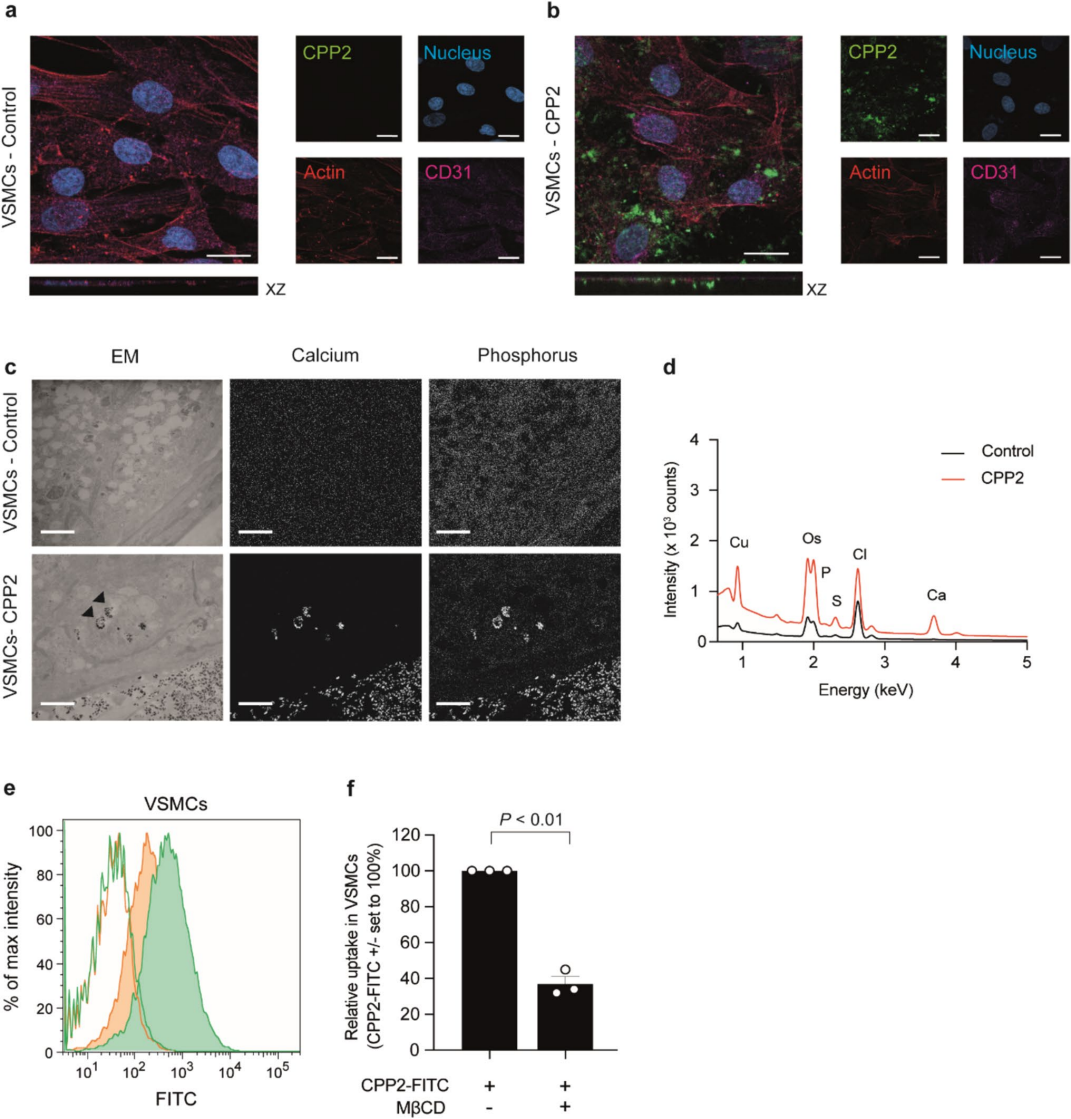
Uptake of CPP2 by VSMCs in vitro. Confocal images of VSMCs (**a**, **b**) treated without (**a**) or with (**b**) FITC-labelled CPP2 (green). **a**, **b** XZ planes show internalization of CPP2 (25 µg/ml) by VSMCs. Nucleus = blue, actin skeleton = red and CD31 = pink (negative in VSMCs). Scale bars represent 20 μm. **c** Electron microscopical analysis of VSMCs showing CPP2 uptake. CPP2-containing vesicles are marked with an arrowhead. Scale bars represent 2 μm. **d** EDX spectra showing copper (Cu), osmium (Os), phosphate (P), sulfur (S), chloride (Cl) and calcium (Ca) for unstimulated VSMCs (control, black line) and the CPP2 stimulated VSMCs (red line). **e**, **f** Flow cytometric analysis quantified the uptake of FITC-labelled CPP2. Endocytosis of CPP2 (green) was inhibited by 2.5 mM methyl-β-cyclodextrin (MβCD; orange) in VSMCs. Lines indicate incubation at 4 °C (extracellular binding), filled histograms indicate incubation at 37 °C (endocytosis + extracellular binding). Data is presented as mean ± SEM of n = 3 individual experiments (each consisting of 3 biological replicates). One sample t-test against a hypothetical value of 100%, P < 0.05 considered statistically significant

Interactions between VSMCs and CPP2 were also investigated with confocal microscopy and fluorescently labelled CPP2. Similar to ECs, labelled CPP2 were also detected intracellularly in VSMCs using confocal microscopy (Fig. 3a, b). Presence of CPP2 in the cytoplasm was demonstrated with electron microscopy (EM) combined with EDX to define the subcellular localization of internalized CPP2. CPP2-resembling particles were detected in cytoplasmic vesicles (marked with arrowheads, Fig. 3c). EDX analysis of these particle-containing vesicles (Fig. 3c,d ) demonstrated the presence of calcium in only the CPP2-exposed cells, confirming the CPP2 identity of the particles in VSMCs similar to ECs. For quantification of uptake of CPP2, VSMCs were incubated with FITC-labelled CPP2 and flow cytometric analysis was performed (Fig. 3e, filled green histogram and Supplementary Fig. S2d(**_LIFE-D-24-02586_R1**)). Incubation of VSMCs with CPP2 in hypometabolic (4 °C) conditions (Fig. 3e, line histograms) resulted in absence of fluorescent signal reflecting no CPP2 uptake. Comparison of CPP2 uptake between ECs and VSMCs demonstrate that ECs internalized more CPP2 than VSMCs (Supplementary Fig. S2e(**_LIFE-D-24-02586_R1**)). Endocytosis inhibitor MβCD reduced CPP2 uptake by 63% in VSMCs (Fig. 3e, filled orange histogram and 3f, *P* < 0.01). Inhibitor cytochalasin D resulted in a similar pattern as it reduced endocytosis with 41% in VSMCs (Supplementary Figure S2g(**_LIFE-D-24-02586_R1**)).

### Conditioned medium of activated ECs enhances VSMC calcification and alters VSMC gene expression

**Fig. 4:**
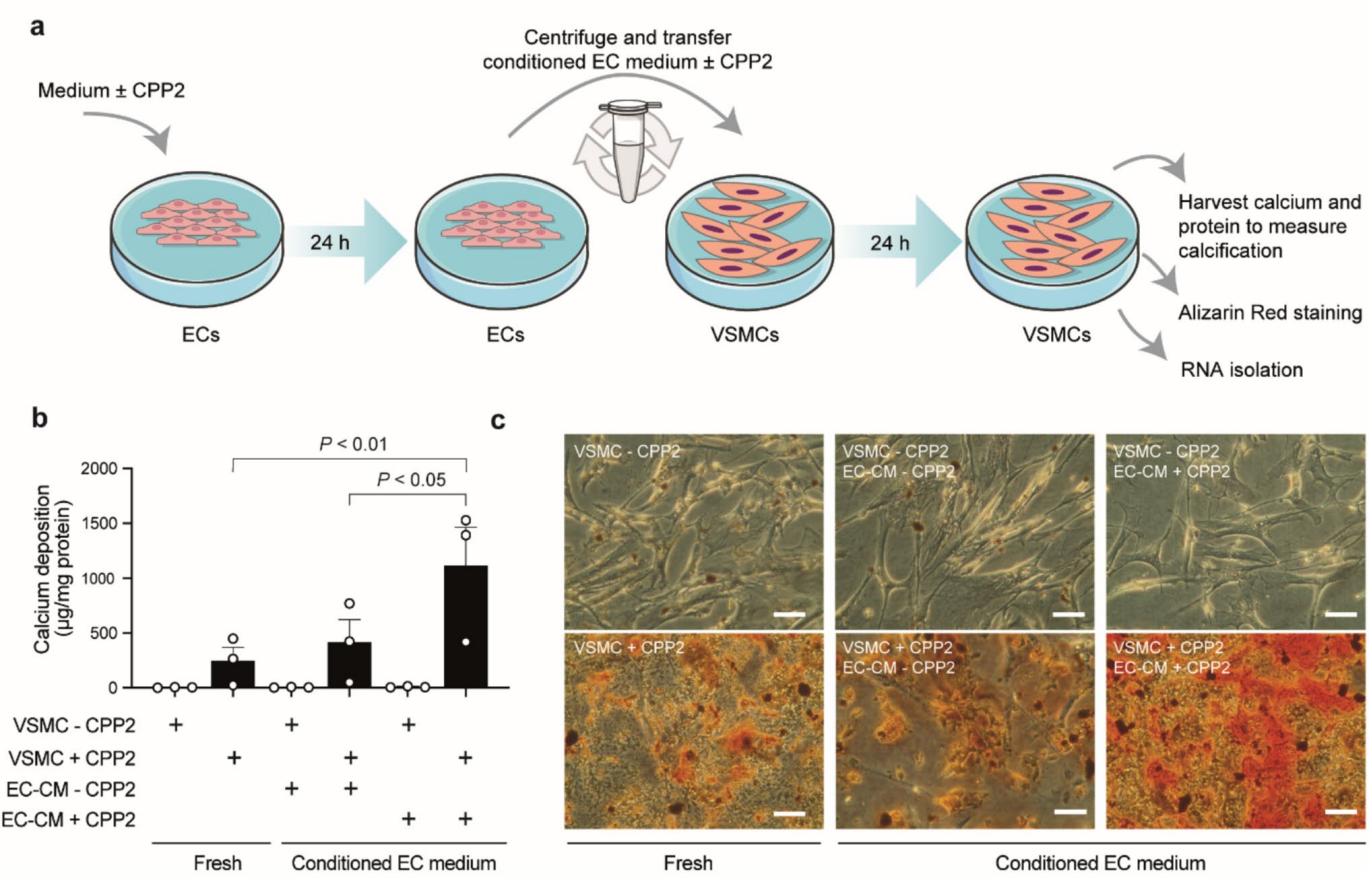
Conditioned medium of activated ECs increased calcification in VSMCs.** a** Schematic overview of conditioned EC medium model. Briefly, ECs were incubated for 24 h with or without 25 µg/ml CPP2. After 24 h the medium from ECs was collected (“EC-CM + CPP2” and “EC-CM – CPP2”) and centrifuged to remove remaining CPP2. Next, the conditioned medium was added to VSMCs in the absence (“VSMC – CPP2”) or presence (“VSMC + CPP2”) of 25 µg/ml CPP2. VSMCs were incubated for 24 h after which calcium deposition was measured and RNA was isolated. **b** VSMCs calcium deposition measured after culture in fresh VSMC medium with or without 25 µg/ml CPP2 (“VSMC + CPP2” and “VSMC – CPP2”, respectively) only, or after culture in EC conditioned medium (“EC-CM – CPP2” and “EC-CM + CPP2”) for 24 h. EC conditioned medium (EC-CM) was derived from ECs exposed to CPP2 or not, as schematically depicted in panel (**a**). Medium from CPP2-activated ECs (“EC-CM + CPP2” induced higher calcification levels than medium from unstimulated ECs (“EC-CM – CPP2”). Data is presented as mean ± SEM of n = 3 individual experiments (each consisting of 3 biological replicates). One way ANOVA followed by Sidák specific multiple comparisons test and *P* < 0.05 was considered statistically significant. **c** Representative Alizarin Red staining visualises calcium depositions in VSMCs under various conditions. Scale bars represent 100 μm

**Fig. 5:**
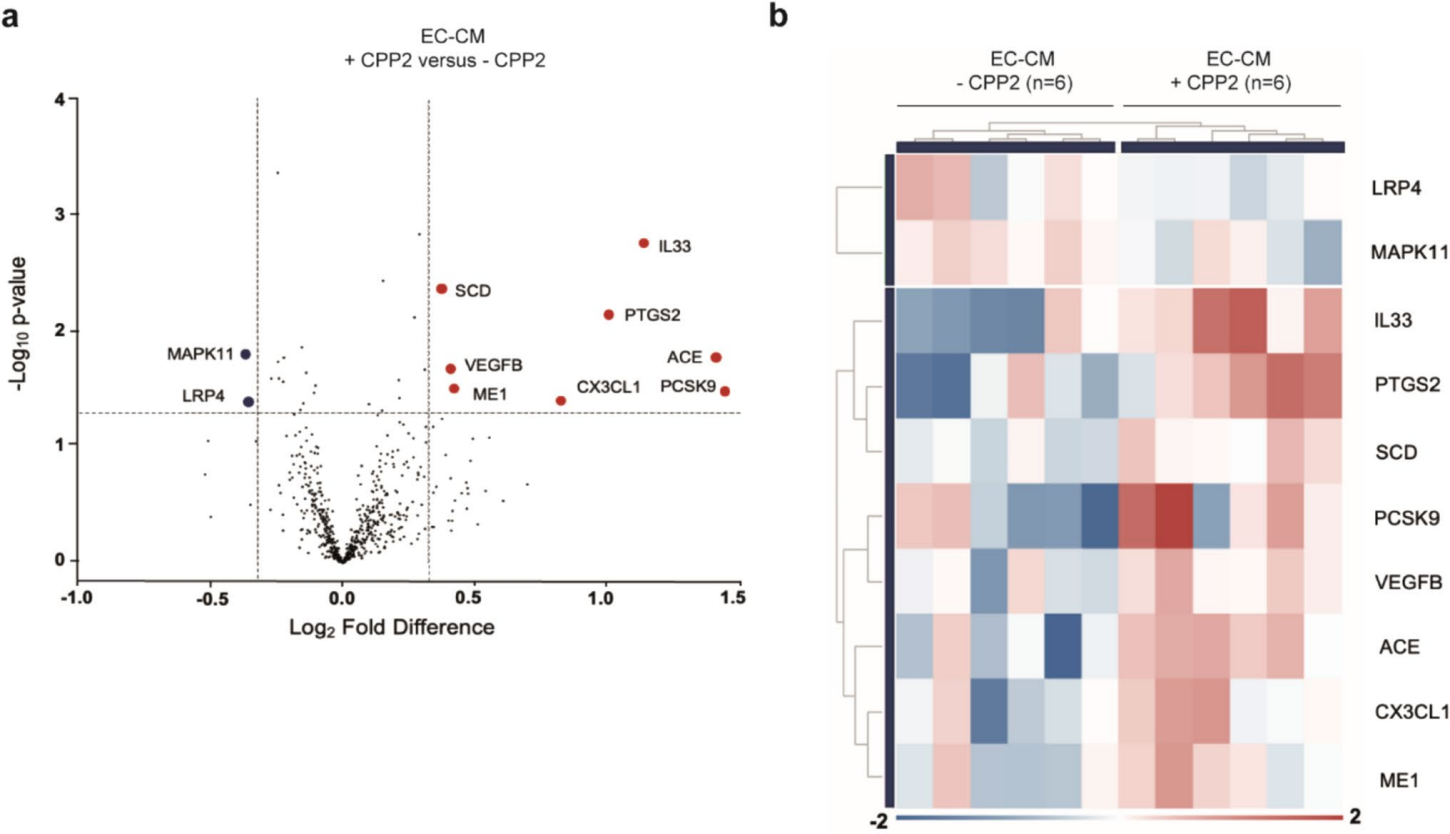
VSMCs exposed to CPP2-activated ECs conditioned medium display differential gene expression patterns. **a** Volcano plot of NanoString nCounter gene expression analysis data indicating significantly upregulated (red dots) and downregulated (blue dots) genes in VSMCs that were cultured in conditioned medium from HUVECs exposed to 25 µg/ml CPP2 (“EC-CM + CPP2”) *vs.* VSMCs cultured in conditioned medium from HUVECs not exposed to CPP2 (“EC-CM – CPP2”) (fold change ≤ – 1.25 or ≥ 1.25 and *P* < 0.05), **b** Heatmap displaying clustering of the “EC-CM -CPP2” and “EC-CM + CPP2” conditioned medium exposed VSMC samples based on the significantly differentially expressed genes

Next, the effect of CPP2-induced EC activation on VSMC calcification was studied in a conditioned medium model (Fig. 4a). Hereto, ECs were incubated for 24 h with 25 CPP2, after which the medium was transferred to a VSMC culture. In between medium transfer, remaining CPP2 from the EC conditioned medium were removed, and depending on the condition, fresh CPP2 were added to the VSMC culture. Calcium deposits were only present in cultures with direct exposure of VSMCs to CPP2 (“VSMC + CPP2”, Fig. 4b, c). Interestingly, when VSMCs were incubated with medium from CPP2-activated ECs (“EC-CM + CPP2”), the calcium deposition of VSMCs was significantly increased compared to incubation with medium from non-activated ECs (“EC-CM – CPP2”, 1.11 ± 0.35 versus 0.41 ± 0.21 mg calcium/mg protein, *P* < 0.05) or VSMCs cultured in non-conditioned “fresh” VSMC medium (0.25 ± 0.12 mg calcium/mg protein, *P* < 0.01). Furthermore, exposure of VSMCs (“VSMC + CPP2”) to EC conditioned medium resulted in alterations in the VSMC transcriptome as assessed with a NanoString nCounter analysis. Ten genes were differentially expressed in VSMCs cultured in the presence of conditioned medium of CPP2-activated ECs (“EC-CM + CPP2”) when compared to VSMCs cultured in conditioned medium from ECs that were not exposed to CPP2 (“EC-CM—CPP2”) (Fig. 5a). Among these 10 genes, pro-calcification genes *PCSK9, CX3CL1, IL33* and *SCD* were significantly upregulated and calcification-inhibitor *LRP4* showed a decreased expression (Fig. 5a,b, Supplementary Table 2, (**LIFE-D-24-02586_R1**) ).

### Secretome analysis of CPP2-induced ECs

**Fig. 6:**
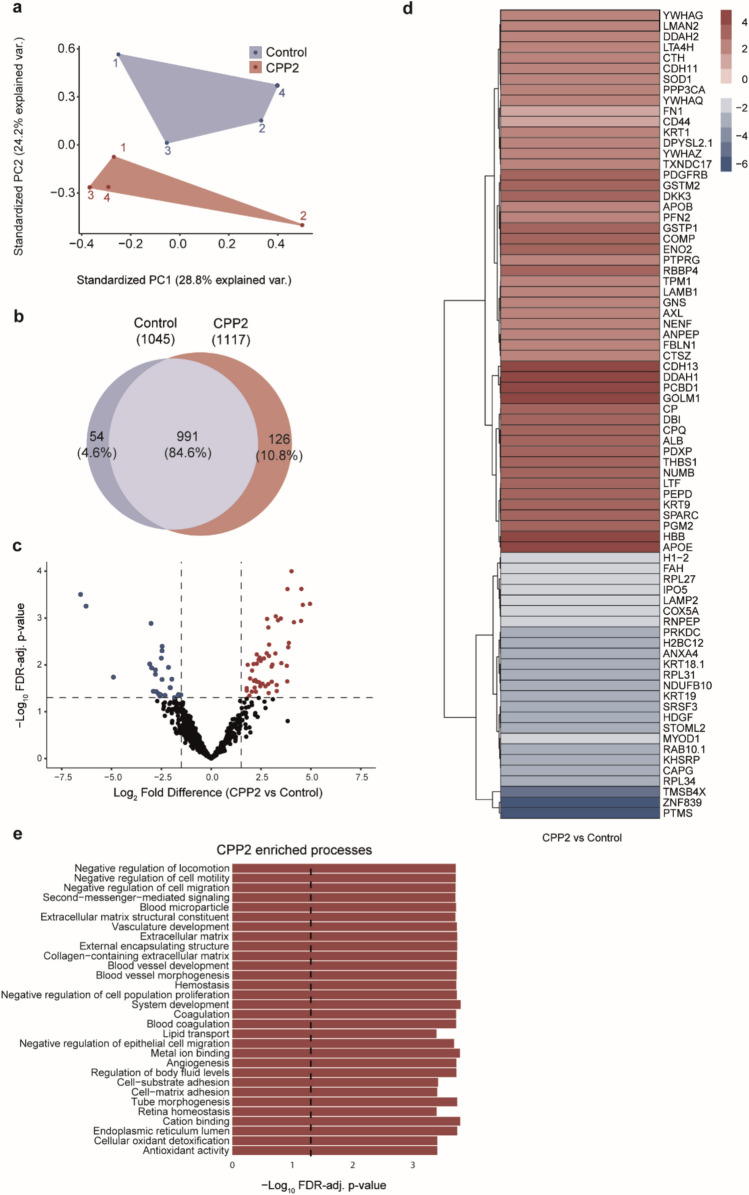
Secretome analysis of conditioned ECs medium. **a** Principal component analysis (PCA) showing the first two components that explain the variance between CPP2-exposed and non-exposed ECs samples. **b** Venn diagram showing overlapping (n = 991 proteins) and differentially secreted proteins (n = 180 proteins) in non-exposed (control) and CPP2-exposed ECs. **c** Volcano plot revealing 76 significantly differentially expressed proteins in medium from CPP2-activated versus unstimulated ECs with cut-off values of FDR-adjusted *P*-value < 0.05 and Log_2_ fold change > 1.5. Significantly increased and decreased proteins are indicated in red and blue, respectively. **d** Heat-map indicating Log_2_ fold change protein expression of the 76 identified proteins. **e** Gene set enrichment analysis (GSEA) of Gene Ontology (GO) terms using ClusterProfiler including the differentially expressed (increased) proteins as shown in panel *d*. Displayed are the top-30 enriched processes based on the CPP2 secretome

To identify the EC-derived factors that enhance VSMC calcification, proteome analysis was performed on albumin-depleted supernatants from ECs, which is indicated as ‘secretome analysis’ in this study. Principal component analysis (PCA) demonstrated that unstimulated ECs samples clustered differently from the CPP2-induced ECs secretome (Fig. 6a). Primary analysis detected a total of 1171 proteins in the supernatant of CPP2-activated ECs (Fig. 6b). Of these 1171 proteins, 991 proteins (84.6%) were also detected in the secretome of the unstimulated (control) ECs (Fig. 6b). The secretome of CPP2-activated ECs contained 126 proteins (10.8%) that were exclusively detected after CPP2-exposure (Fig. 6b, Supplementary Table 3 (**exclusiveCPP25**)), and 54 proteins (4.6%) from which reads were exclusively detected in the unstimulated ECs (Fig. 6b, Supplementary Table 4 (**exclusiveControl**)). Using a FDR-adjusted *P*-value < 0.05 and Log_2_ Fold Change > 1.5, 76 differentially expressed proteins were identified in the secretome of CPP2-activated ECs versus unstimulated ECs (Fig. 6c, red and blue dots). Data underlying the secretome analysis can be found in Supplementary Table 6 (**RawData_Secretome**). Expression ratios between unstimulated and CPP2-activated EC-derived secretome are shown in the corresponding heat-map (Fig. 6d). Subsequently, gene-set enrichment analysis (GSEA) using gene ontology (GO) terms and Kyoto Encyclopedia of Genes and Genomes (KEGG) pathways was performed. When using GO-terms, many different processes were significantly enriched upon CPP2 exposure (Supplementary Table 5 (**GSEA**)), of which the top 30 CPP2-enriched processes are shown in Fig. 6e. These processes include blood vessel development, extracellular matrix remodeling, and oxidative stress-related processes. Also, various processes were significantly enriched in unstimulated (control) ECs (*i.e.*, downregulated upon CPP2 exposure, Supplementary Table 5 (**GSEA**)), of which the top 30 control-enriched processes are shown in Supplemental Fig. S4. CPP2 exposure resulted in downregulation of processes related to RNA handling and translation (Supplemental Figure S4). GSEA analysis using KEGG pathways also resulted in significantly enriched processes (Supplementary Table 5 (**GSEA**)), of which some were related to the processes observed using GO-terms such as “Ribosome”, “ECM-receptor interaction”, “Cell adhesion molecules”, and “Focal adhesion”. Other processes were more general in nature (e.g. “Oocyte meiosis”, “MicroRNAs in cancer”, “Alcoholism”) and harder to link to processes occurring in endothelial cells when compared to GSEA based on GO-terms.

## Discussion

In the present study, we investigated the paracrine signaling between ECs and VSMCs in the context of CPP2-induced VSMC calcification. We demonstrated that CPP2-activated ECs enhance VSMC calcification in a conditioned medium model. The secretome of CPP2-activated ECs contained proteins related to blood vessel development, extracellular matrix remodeling, and oxidative stress-related processes. Moreover, in response to the conditioned EC-medium, VSMCs upregulated pro-calcification markers. Altogether, these results demonstrate that CPP2-induced EC activation enhances VSMC calcification in a paracrine manner.

The main finding of this paper is that CPP2-induced EC activation affects VSMC function. This is in line with previous studies demonstrating paracrine effects of ECs on VSMCs in a variety of vascular diseases [4, 32, 33]. VSMC phenotype and function can be altered by ECs, via the secretion of paracrine factors (*e.g.*, cytokines, growth factors and hormones) or through the release of EC-derived extracellular vesicles (EVs) [4, 32, 33]. In the current study, we show that culturing VSMCs in conditioned medium derived from CPP2-activated ECs significantly enhanced calcium deposition on the VSMCs, indicating that ECs modulate CPP2-induced VSMC calcification in a paracrine manner. In a study by Shishkova et al., conditioned medium of CPP2-exposed human coronary artery endothelial cells (HCAEC) led to a pro-inflammatory activation of naive HCAEC. [34] In addition to these observations, we now show that this CPP2-derived EC-secretome has also a pro-calcifying nature, which not only gives insight into the response of ECs to CPP2 but also provides molecular understanding of the role of ECs in CPP2-induced VSMC calcification.

The mass spectrometry analysis of the EC-derived secretome revealed 76 proteins which were differentially expressed in the supernatant of CPP2-stimulated ECs when compared to non-stimulated control ECs. Of these, 51 and 25 proteins were significantly upregulated and downregulated, respectively. Although it is tempting to focus on the individual proteins from the secretome as candidates being responsible for enhanced calcification, it is most likely that the combined action of multiple proteins explains the pro-calcifying properties on VSMCs. Previous studies have demonstrated that the EC secretome changes upon pathogenic stimulations, resulting in pleiotropic downstream effects [35–37]. To gain further insight in alternative mechanisms by which CPP2 uptake causes cellular damage, paracrine signaling, and subsequent VSMC calcification, we performed gene-set enrichment analysis on the EC-derived secretome after CPP2 exposure. Interestingly, extracellular matrix pathways were significantly enriched in the CPP2-activated EC secretome. Matrix remodeling is well established as main contributor to vascular calcification in CKD [4, 38]. In particular, matrix metalloproteases have been implicated in elastin degradation but also in guiding the phenotypic transformation of VSMCs [39, 40]. Significantly upregulated proteins in these pathways included fibronectin (FN1) and aggrecan (ACAN). Indeed, fibronectin deposition has been associated with increased calcification of vascular cells [41] and aggrecan has been demonstrated to be present in calcified atherosclerotic plaques [42]. In contrast to our data Stepanov et al. recently demonstrated reduced abundance of FN1 in their secretome analysis, whereas CD59 was increased [43]. We did not detect CD59 (protectin) in our dataset. The cause of this discrepancy between both studies is as yet unknown. In line with our in vitro secretome data on extracellular matrix remodeling, we recently demonstrated that vascular remodeling processes, including extracellular matrix remodeling, in vascular biopsies of CKD patients are associated with increased circulating CPP counts. [30] Whether the pro-calcifying nature of the CPP2-derived EC-secretome also includes EV-related signaling, cannot be excluded. Although, the secretome data did not contain classical EV markers such as CD9, CD63 and CD81, conditioned media from this study were not subjected to ultracentrifugation, which is usually required to remove EVs [44]. Therefore, future studies might focus on the possible contribution of EVs in enhanced calcification as observed in our conditioned medium model.

In addition, our data demonstrated uptake of CPP2 by ECs using confocal and electron microscopy. Due to the acidic environment present in lysosomes, crystalline CPP2 can dissociate into soluble calcium and phosphate when CPP2-rich endosomes mature into lysosomes. Dissociation of CPP2 might subsequently lead to increased intracellular calcium and phosphate levels [45]. Indeed, calcium and phosphate overload can lead to increased ROS production, EC dysfunction and apoptosis [46, 47]. As oxidative distress is one of the main drivers of ECM remodeling and initiation of vascular calcifications[48, 49], it could be speculated that this is the underlying mechanism. Future studies should determine the effects of oxidative distress on the EC secretome however based on GSEA we here already demonstrated that *Cellular oxidant detoxification* and *Antioxidant activity* processes were enriched in the CPP2 secretome.

We furthermore showed that in response to CPP2 exposure ECs (both HUVECs and HAECs) get activated as demonstrated by increased ICAM-1, VCAM-1 and E-selectin mRNA expression, which supports data reported by others [22–24]. We previously showed that this activation is also associated with the development of endothelial dysfunction as exposure to CPP2 impaired eNOS signaling and vasomotor activity as well as mitochondrial ROS production and peroxynitrite-mediated protein damage [25]. Effects hereof on the altered secretome is therefore not unlikely. Whether this causality between CPP2 exposure and EC activation and dysfunction also exists in humans in vivo remains to be demonstrated. However, we here demonstrated a significant correlation between CPP1 and CPP2 counts and the endothelial dysfunction marker sVCAM-1 in a cohort of healthy kidney donors and CKD patients. These data are in line with our previous in vitro data[25], and suggest that also in the clinical setting in vivo CPP counts are associated with EC activation and dysfunction.

An interesting observation was the absence of an osteochondrogenic dedifferentiation expression profile after exposure of VSMC to CPP2 for 24 h. Vascular calcification is a complex process that includes both passive mineral deposition and active cellular calcification involving osteochondrogenic VSMC dedifferentiation [50]. CPP2 are generally considered to be involved in vascular calcification initiation, which depends on initial mineral deposition and cellular damage. Our timeframe of 24 h CPP2 exposure most likely mimics the initiation phase of calcification. For VSMC dedifferentiation presumably a longer exposure time is required. Furthermore, it is still a matter of debate whether osteogenic VSMC dedifferentiation is an absolute prerequisite for vascular calcification in CKD as recent studies also have shown that VSMC might calcify in the absence of the classical dedifferentiation process. [30]

Finally, we performed NanoString nCounter expression analysis on VSMCs that were exposed to EC-derived conditioned medium, to map the consequences of the CPP2-activated EC secretome on VSMCs. Out of ten differentially expressed genes, six genes were previously linked to calcification. The gene that was most differentially expressed is *PCSK9*, which encodes an enzyme involved in lipoprotein metabolism. Indeed, increased levels of PCSK9 have been associated with calcification [51]. Although primarily expressed in the liver, PCSK9 is also expressed in VSMCs and has been associated with VSMC senescence and apoptosis [52]. Direct effects of VSMC-derived PCSK9 on calcification in the setting of CKD were demonstrated [53]. Subjects with CKD and atherosclerotic calcification were characterized by increased levels of circulating PCSK9. Overexpression of PCSK9 in VSMCs, but not exogenous recombinant PCSK9, enhanced calcification. Furthermore, the inflammatory mediators IL-33 and CX3CL1 were shown to be significantly upregulated in VSMCs exposed to CPP2-activated conditioned medium as well. IL-33 is a pro-inflammatory cytokine belonging to the IL-1 family. Although literature on the role of IL-33 in calcification is scarce, patients suffering from nonrheumatic aortic valve stenosis (with calcified lesions in aortic valves) were shown to have increased plasma IL-33 levels [54]. Furthermore, valvular interstitial cells exposed to IL-33 in vitro were shown to upregulate pro-calcifying factors in a ST2-dependent manner [54]. Valvular interstitial cells were also shown to upregulate endogenous IL-33 expression in vitro upon stimulation as well as in aortic valve stenosis tissue [55]. Altogether, these findings demonstrate that VSMCs obtain a more pro-calcification expression pattern in response to CPP2-activated EC conditioned medium. This altered gene expression pattern was not associated with enhanced VSMC apoptosis based on the absence of differential expression within 35 apoptosis-related genes that were included in the NanoString nCounter CVD Pathophysiology panel (not shown).

In conclusion, CPP2-activated ECs promote VSMC calcification via paracrine signaling. In response to these paracrine factors, VSMCs increase the expression of pro-calcification genes. Therapeutic interventions to prevent CPP2-induced EC activation could reduce pro-calcifying paracrine signaling between ECs and VSMCs, and attenuate VC in CKD.

## Materials & methods

### Clinical sample collection

Clinical plasma and serum samples were obtained within a sub study of the *TransplantLines* biobank cohort, which is a large longitudinal cohort study investigating various outcomes after organ transplantation of patients in the University Medical Center Groningen (UMCG) [56]. Plasma and serum of 17 healthy living kidney donors and 34 kidney transplant recipients (CKD patients) were included in this study. The CKD group comprised both pre-emptive dialysis and dialysis CKD patients.

### Soluble VCAM-1 (sVCAM-1) ELISA

sVCAM-1 levels were measured in plasma using the Human VCAM-1/CD106 ELISA kit (#DY809-05, R&D systems, USA), according to manufacturer’s protocol. In brief, plates were overnight coated with the capture antibody. Samples were diluted 1:1000 using Reagent Diluent. Diluted samples (100 µL) or standards were added to the plates for 2 h room temperature (RT). After incubation, samples were aspirated, and wells were washed with Wash Buffer. The detection antibody (100 µL) was incubated for 2 h RT. Subsequently, the detection antibody was aspirated and washed away with Wash Buffer. Streptavidin-HRP D working solution (100 µL) was added for 20 min in the dark RT and washed away afterwards. Next, plates were incubated for 20 min with Substrate Solution (100 µL) per well at RT in the dark. Immediately thereafter, 50 µL of Stop Solution was added. The optical density (OD) was measured at 450 nm and corrected for background signal at 540 nm. sVCAM-1 levels are expressed in µg/ml.

### CPP1 and CPP2 quantification

CPP levels (CPP1 and CPP2 counts) were quantified according to our previously published methods[57, 58], with minor modifications as recently published[30] and described below. All solutions were twice 0.22 µm-filtered prior to use. Bovine fetuin-A was obtained from Sigma (#F3004, Sigma-Aldrich, USA) and the monomer purified by size exclusion chromatography as described previously [11]. Frozen serum samples were thawed in a water bath at 37 °C with gentle agitation. Five µL portions were mixed with 40 µL HEPES-buffered DMEM (50 mM HEPES, no phenol red, pH 7.45) and 5 µL staining solution containing Alexa Fluor 647-conjugated risedronate (5 µM, #BV500101, BioVinc, USA), FITC-conjugated lactadherin (1.5 µg/mL, #BLAC-FITC, Haematologic Technologies Inc., USA) and mFluor violet 450-labelled bovine fetuin-A (2 µg/mL, prepared in-house using the ReadiLink™ Rapid mFluor™ Violet 450 Labeling Kit, #1100, AAT Bioquest, USA). After 120 min incubation in the dark with gentle mixing, samples were diluted to 500 µL in HEPES-buffered DMEM. Measurements were acquired in triplicate using a calibrated Apogee A50/Micro flow cytometer equipped with 50mW 405 nm, 488 nm and 638 nm lasers (parameter settings: sheath pressure 150 mbar, four flush cycles). Flow rate (3 µL/min) and measurement times (120 s or until data storage buffer was full – 5.000.000 events) were held constant for all samples. Fluorescence threshold triggering was used to detect fetuin-A-stained particles and a negative gating strategy was used to exclude membrane-delimited particles which stain positive for FITC-conjugated lactadherin. CPPs were gated as fetuin-A^+^ lactadherin^−^ events. Amorphous calcium phosphate-containing and crystalline hydroxyapatite-containing populations were gated based on their differential affinity for risedronate: risedronate^LO^ (CPP1) and risedronate^HI^ (CPP2), respectively. Samples positive for gross lipidaemia or haemolysis were excluded. Sample sizes were based on the availability of serum.

### CPP synthesis

Secondary calciprotein particles (CPP2) were generated in phenol red-free Dulbecco's Modified Eagle Medium (DMEM) (Gibco, ThermoFisher Scientific, USA) supplemented with 3.5 mM phosphate (NaH_2_PO_4_), and 1 mM calcium (CaCl_2_), 10% (v/v) fetal bovine serum (FBS) and 1% (v/v) penicillin/streptomycin. After 14 days of incubation at 37 °C, CPP2 were isolated by two rounds of ultracentrifugation for 2 h at 24,000 g at 4 °C, including a washing step with 1 × TBS. CPP2 were used for experiments within 60 days of isolation, and stored in TBS at 4 °C. The CPP2 concentration was determined based on the calcium content measured with the calcium colorimetric assay kit (#MAK022, Sigma-Aldrich, USA) or o-cresolphthalein calcium assay method [59]. For the in vitro experiments, cells were exposed to either 0 μg calcium/ml (= control, no CPP2) or a volume equivalent to 25 μg calcium/ml (= 25 CPP2) for 24 h.

### Cell culture

Human Umbilical Vein Endothelial Cells (HUVECs) were purchased from Lonza (#CC-2519, Switzerland) and cultured in endothelial cell culture medium (ECM; RPMI-1640 basal medium (Lonza, Switzerland) supplemented with 20% (v/v) FBS, 1% (w/v) L-glutamine, 1% (v/v) penicillin/streptomycin, 50 mg/L ECGF (homemade isolation)[60] and 5 U/ml heparin, as described previously [61]. Cells were grown on 1% (w/v) gelatin-coated culture dishes and incubated at 37 °C with 5% (v/v) CO_2_. HUVECs were expanded and used for experiments between passage 3 and 7 at 80% confluence (20,000 cells/cm^2^).

Human Aortic Endothelial Cells (HAECs) were purchased from ThermoFisher Scientific (#C-006-5C, USA) and cultured in Human Large Vessel Endothelial Cell Basal Medium (#M-200–500, ThermoFisher Scientific, USA) supplemented with Cascade Biologics Low Serum Growth Supplement (LSGC) 50x (#S-003–10, Gibco, ThermoFisher Scientific, USA). Cells were grown in plastic culture dishes incubated at 37 °C with 5% (v/v) CO_2_. HAECs were expanded and used for experiments between passage 4 and 7 at confluence of 80% (20,000 cells/cm^2^).

Human Aortic Smooth Muscle Cells (HASMCs) were purchased from the American Type Culture Collection (#PCS-100–012, ATCC, USA). VSMCs were expanded in DMEM medium (Lonza, Switzerland) supplemented with 10% (v/v) FBS, 2 mM L-glutamine, 0.1 mM non-essential amino acids (ThermoFisher Scientific, USA), 10 ng/ml EGF and 5 µg/ml insulin (Sigma-Aldrich, USA). During calcification experiments, VSMCs were cultured in the same medium without the supplementation of EGF and insulin. Cells were kept in a 37 °C incubator with 5% (v/v) CO_2_. Passages between 6 and 10 were used for experiments.

### Culture of VSMCs in conditioned medium

For the conditioned medium experiments, ECs and VSMCs were cultured separately. ECs were stimulated without CPP2 (= control) or with 25 CPP2 for 24 h at 37 °C with 5% (v/v) CO_2_ in M199 medium (Lonza, Switzerland) supplemented with 5% (v/v) FBS, 2 mM L-glutamine, 0.5 U/ml heparin and 25 µg/ml bovine pituitary extract (BPE, ThermoFisher Scientific, USA). After 24 h, medium was collected and centrifuged for 30 min at 10,000 g to remove CPP2. Subsequently, either new CPP2 (volume equivalent to 25 μg Ca^2+^/ml medium) or no CPP2 (TBS volume control) were added to the conditioned medium. The medium was added to the VSMCs for 24 h at 37 °C with 5% CO_2_ (v/v). After 24 h, VSMC total RNA was isolated to assess gene expression and calcification was determined (both described below).

### Gene expression analysis using RT-qPCR

Total RNA was extracted from the ECs (HUVECs and HAECs) and VSMCs with TRIzol Reagent (ThermoFisher Scientific, USA) according to manufacturer’s protocol. Briefly, cells were lysed in TRIzol and RNA was separated from the genomic DNA and organic components by adding chloroform. RNA was precipitated with isopropanol and washed with 70% (v/v) of ethanol. The RNA quantity and purity were measured with the Nanodrop spectrophotometer 1000 (ThermoFisher Scientific, USA). Next, cDNA was synthesized with the MLV reverse transcriptase kit or Superscript II Reverse Transcriptase (ThermoFisher Scientific, USA), according to the manufacturer’s protocol. For RT-qPCR analysis, 5 ng of cDNA was combined with SYBR green reaction mixture (Roche, Switzerland) and 10 µM of forward and reverse primers (Supplementary Table 1 (**LIFE-D-24-02586_R1**)). Reactions were performed on the LightCycler 480 system (Roche, Switzerland) or CFX96 connect Real time system (Bio-rad, USA). Off-target amplification was assessed by analyzing melt curves and led in this case to exclusion of the respective sample from the dataset. Gene expression was calculated relative to β2M housekeeping gene expression (2^−ΔCt^) and expressed as fold-change compared to the experimental control (set to 1).

### Gene expression analysis using NanoString nCounter

Quantitative gene expression analysis was performed on RNA isolated from VSMCs using the NanoString nCounter platform. Hereto, VSMCs were cultured for 24 h (in the presence of 25 CPP2) in conditioned medium derived from HUVECs that were stimulated with (+ CPP2) or without (no CPP2) 25 CPP2, also for 24 h. For each condition 6 individual VSMC samples were included. One hundred ng of total RNA was hybridized for 16 h at 65 °C to a core set of Reporter probes, using the nCounter CVD Pathophysiology Panel (including 800 CVD-related genes) (NanoString Technologies, USA) and Capture probes. Digital data collection of the hybridized mixture was performed using the Prep Station and Digital Analyzer of the nCounter FLEX analysis system. The cartridge was scanned 555 field of view to identify the unique barcodes. Raw data were analysed using nSolver 4.0 and NanoString ROSALIND (version 3.37.6.11). Thresholds were set at fold change ≤ -1.25 or ≥ 1.25 and *P* < 0.05.

### Quantification of calcification deposits

VSMCs were decalcified with 0.1 M HCl and extracellular calcium deposition was quantified using the o-cresolphthalein calcium assay described previously [59]. To correct for the number of cells, VSMCs were solubilized with 0.1 M NaOH containing 1% (w/v) SDS in which protein content was determined using the BCA kit (ThermoFisher Scientific, USA) according to the manufacturer's protocol.

### Alizarin Red staining

To visualize calcification, cells were fixed using 4% (w/v) buffered paraformaldehyde (VWR) for 15 min and subsequently stained using an aqueous 2% (w/v) Alizarin Red solution pH 5.4 (Sigma-Aldrich, USA) for 5 min. Excess dye was removed by washing one to two times with Milli-Q water.

### Fluorescent labelling of CPP2

CPP2 were labelled with fluorescein isothiocyanate (FITC, Sigma-Aldrich, USA) according to the manufacturer's protocol with minor changes. To replenish the storage solution with buffer required for labelling, CPP2 were centrifuged twice for 30 min at 18,000 g at 4 °C and resuspended in freshly made 0.1 M sodium carbonate pH 9. Subsequently, 0.05 mg FITC was added per mg protein (as measured with the BCA kit) to the CPP2 solution and incubated overnight at 4 °C. Thereafter, ammonium chloride (NH_4_Cl) was added to a final concentration of 50 mM and incubated for 2 h at 4 °C. Unbound FITC was removed by two rounds of centrifugation of the CPP2 at 18,000 g at 4 °C. In between centrifugation steps CPP2 were resuspended in TBS.

### Fluorescence microscopy

Endocytosis of CPP2 by the ECs or VSMCs was visualized by fluorescence microscopy. Cells were grown on glass coverslips and treated with or without FITC-labelled CPP2 for 24 h at 37 °C and 5% (v/v) CO_2_. Cells were fixed in 4% (w/v) paraformaldehyde in PBS (ThermoFisher Scientific, USA) for 10 min after which they were permeabilized in 0.3% (v/v) Tween-20 and 0.1% (w/v) bovine serum albumin (BSA) in PBS for 10 min, followed by a 10-min incubation with 50 mM NH_4_Cl in PBS to quench free aldehyde groups. Next, non-specific protein binding was blocked by goat serum dilution buffer (GSDB) containing 16% (v/v) goat serum and 0.3% (v/v) Tween-20 for 30 min before incubation with the primary antibody for the membrane marker CD31 (1:25, #ab28364, rabbit-anti-human, Abcam, UK) diluted in GSDB overnight at 4 °C. Secondary antibody (1:300, #A21245, Alexa Fluor 647 goat-anti-rabbit, ThermoFisher Scientific, USA) in GSDB was incubated together with phalloidin (1:400, #A12381 Alexa Fluor 594, ThermoFisher Scientific, USA) and DAPI (1:1,000, #D1306, Life technologies, USA) for 45 min at RT. Coverslips were mounted with Fluoromount G (Southern Biotech, USA) and stored at 4 °C until analysis. Images were obtained with a LSM900 confocal laser scanning microscope (Zeiss, Germany) and processed with Fiji software version 2.3.0.

### Electron microscopy (EM)

After a 20 h stimulation with CPP2, ECs and VSMCs were fixed by adding dropwise an equal volume of fixative (2% (w/v) glutaraldehyde plus 2% (w/v) paraformaldehyde in 0.1 M sodium cacodylate buffer) to the culture medium. After 10 min, the mixture was replaced by pure fixative and incubated for 30 min at RT. Cells were post fixated in 1% (v/v) osmium tetroxide/1.5% (w/v) potassium ferrocyanide (30 min at 4 °C) and dehydrated using ethanol and embedded in EPON epoxy resin. Sections (100 nm) were cut and collected on single slot copper grids with formvar support film. Images were taken with a Zeiss Supra55 in STEM mode at 28 kV using an external scan generator (Fibics, Canada) yielding mosaics of large area scans at 2.5 nm pixel resolution as described in detail [62]. Large scale TIF images were stitched and converted to html files using Atlas software (Fibics, Canada). The html files can be accessed using the following link for review purposes:

http://www.nanotomy.org/OA/Feenstra2022SUB/. *(Of note: upon acceptance the data is available through *www.nanotomy.org*).*

### Energy-dispersive X-ray spectroscopy (EDX)

Elemental mapping (EDX) was performed using a TalosF200i operated at 80 kV equipped with DualX EDX detectors using Velox software (ThermoFisher Scientific, USA). STEM imaging was done at a camera length of 205 mm with the HAADF detector at a convergence angle 47 – 200 mRad. For elemental mapping a combination of gun lens 4 and spot size 5 yielded a screen current of approximately 2nA, which was the maximum before the sample was drifting too much, and this setting gave a count rate of approximately 15 kcps per detector. In total, 50 -100 frames were collected with 50 µs dwell time.

### Flow cytometric analysis

ECs and VSMCs were incubated for 4 h at either 4 °C or 37 °C with FITC-labelled CPP2 with or without 2.5 mM endocytosis inhibitor methyl-beta-cyclodextrin (MβCD, Sigma-Aldrich, USA) or 1 μM cytochalasin D (Santa Cruz Biotechnology, USA) before trypsinizing the cells. Propidium iodide was added (20 μg/ml final concentration) and the cells were analyzed on a FACSVerse (BD Biosciences, USA) flow cytometer with a measurement of maximum 1 min or when 10,000 events per sample were obtained. Using FlowJo Software v9 (TreeStar Inc., USA) dead cells were identified based on propidium iodide positivity and FSC-A, and excluded from analysis (Supplementary Figure S2a and S2b(**LIFE-D-24-02586_R1**)). Samples which were kept at 4 °C were used as a control for cell surface binding of CPP2. To quantify endocytosis, correction for the extracellularly attached CPP2 was applied by subtracting the absolute geometric mean fluorescence intensity (gMFI) values obtained at 4 °C from the gMFI values obtained at 37 °C.

### Sample collection and preparation for secretome analysis

After 24 h stimulation with CPP2, medium of ECs (HUVECs) was collected and centrifuged for 15 min at 1,300 g and 4 °C to remove cell debris. To reduce the BSA signal (derived from the FBS in culture medium) during secretome analysis, samples were albumin-depleted using the Albumin Depletion Kit (ThermoFisher Scientific, USA). Hereto, the supernatants were purified with resin-containing albumin depletion columns. After purification, columns were washed with 50 µL low-salt binding/wash buffer containing 25 mM Tris, 25 mM NaCl (pH 7.5). In total, 100 µL of purified supernatant was collected and the protein concentration was measured with a protein colorimetric assay kit (Bio-rad, USA). Samples were stored at -20 °C prior to the secretome analysis.

### Secretome analysis

For the secretome analysis, 2.5 µg protein extract was prepared using a modified Gel-aided Sample Preparation protocol. [63] Samples were digested with trypsin/Lys-C overnight at 37 °C. For nano-LC fragmentation, protein or peptide samples were first desalted and concentrated onto a µC18 Omix (Agilent, USA) before analysis. The chromatography step was performed on a NanoElute (Bruker Daltonics, USA) ultra-high-pressure nano flow chromatography system. Approximately 200 ng of each peptide sample were concentrated onto a C18 pepmap 100 (5 mm x 300 µm i.d.) precolumn (ThermoFisher Scientific, USA) and separated at 50 °C with a reversed phase Reprosil column (25 cm x 75 μm i.d.) packed with 1.6 μm C18 coated porous silica beads (Ionopticks, Australia). Mobile phases consisted of 0.1% (v/v) formic acid, 99.9% water (v/v) (A) and 0.1% (v/v) formic acid in 99.9% ACN (v/v) (B). The nanoflow rate was set at 250 nL/min, and the gradient profile was as follows: from 2 to 30% B within 70 min, followed by an increase to 37% B within 5 min and further to 85% within 5 min and re-equilibration. MS experiments were carried out on an TIMS-TOF pro-mass spectrometer (Bruker Daltonics, USA) with a modified nano electrospray ion source (CaptiveSpray, Bruker Daltonics, USA). A 1400 spray voltage with a capillary temperature of 180 °C was typically employed for ionizing. MS spectra were acquired in the positive mode in the mass range from 100 to 1700 m/z and 0.60 to 1.60 1/k0 window. In current analyses, the mass spectrometer was operated in PASEF DIA mode with exclusion of single charged peptides. The DIA acquisition scheme consisted of 16 variable windows ranging from 400 to 1200 m/z.

### Database searching and protein identification secretome

Database searching and LFQ quantification (using XIC) was performed using DIA-NN (version 1.6.0). An updated UniProt *Homo sapiens* database was used for library-free search/library generation. For RT prediction and extraction mass accuracy, we used the default parameter 0.0, which means DIA-NN performed automatic mass and RT correction. Top six fragments (ranked by their library intensities) were used for peptide identification and quantification. The false discovery rate (FDR) was set to 1% at the peptide precursor level. The variable modifications allowed were as follows: Nterm-acetylation and Carbamylation (RK). In addition, C-Propionamide was set as a fixed modification. “Trypsin/P” was selected. Data were filtered according to a FDR of 1%. Cross-run normalization was performed using RT-dependent. To quantify the relative levels of protein abundance between different groups, data from DIA-NN were further processed in Perseus (v. 1.5.1.0) [64]. Briefly, data were Log_2_ transformed, proteins that were identified in 3 out of 4 replicates of at least one condition were filtered, and remaining missing values were imputed using the default settings (width = 0.3, down shift = 1.8). Downstream analyses were then performed in the program R (v. 4.2.2) with the DEP package (v. 1.20.0) [65]. Differential enrichment analysis was based on linear models and empirical Bayes statistic. A 1.5-fold increase in relative abundance and a 0.05 FDR were used to determine enriched proteins. Finally, the foldchange of all proteins were used for Gene Set Enrichment Analysis (GSEA) of Gene Ontology and KEGG pathways using ClusterProfiler [66, 67].

### Statistics

Statistical analyses were performed with Graphpad Prism 9 (Graphpad Software Inc, USA). Differences between two groups were tested with a Mann–Whitney U test. Differences between three or more experimental groups were tested with one-way ANOVA and Šídák's multiple comparisons test. Inhibition of endocytosis was analysed with an one sample t-test to a hypothetical value of 100%. Correlation between sVCAM-1 and CPP counts was performed using two-tailed nonparametric Spearman correlation. Data is presented as mean ± standard error of the mean (SEM). Statistical analyses on clinical demographics were performed with IBM Statistical Package for Social Sciences (SPSS, version 28). Demographical data is presented as mean ± standard deviation (SD) and tested with unpaired t-test, or median ± interquartile range (IQR) and tested with Mann–Whitney U test (continuous data). Categorical data is tested with Chi-squared test. *P* < 0.05 was considered statistically significant. Graphs were generated with GraphPad Prism 9 and figures created with Adobe Illustrator 27.7 (Adobe Inc., USA).

## Supplementary Information

Below is the link to the electronic supplementary material.

Supplementary file1 (DOCX 1850 KB)

Supplementary file2 (XLSX 30 KB)

Supplementary file3 (XLSX 25 KB)

Supplementary file4 (XLS 363 KB)

Supplementary file5 (XLSX 219 KB)

## Data Availability

The data underlying this article will be shared on request to the corresponding author. The html files of electron microscopic images can be accessed using the following link for review purposes: http://www.nanotomy.org/OA/Feenstra2022SUB/. *(Of note: upon acceptance the data is available through *www.nanotomy.org*).* Raw secretome data are deposited in the public repository iProX (integrated proteome resources center, iProX—integrated Proteome resources, IPX0007030001).
