# Supplementary material for: Calciprotein particle-activated endothelial cells aggravate smooth muscle cell calcification via paracrine signalling
Source: Cell Mol Life Sci. 2025 Apr 26;82(1):177. doi: 10.1007/s00018-025-05702-z (PMC12033162; doi:10.1007/s00018-025-05702-z)
Supplement: Supplementary file 1 — Supplementary file1 (DOCX 1850 KB) [file 18_2025_5702_MOESM1_ESM.docx]

**SUPPLEMENTARY INFORMATION**

**CALCIPROTEIN PARTICLE-ACTIVATED ENDOTHELIAL CELLS AGGRAVATE SMOOTH MUSCLE CELL CALCIFICATION VIA PARACRINE SIGNALLING**

Lian Feenstra,^a^ Lara W. Zeper,^b^ **Brenda van de Langenberg,^b^** Eveline J.E.M. Kahlman,^b^ Guido de La Roij,^b^ Melanie Reijrink,^a^ Benoit Bernay,^c^ Laurent Chatre,^d^ Jeroen Kuipers,^e^ Ben N.G. Giepmans,^e^ Mirjam F. Mastik,^a^ Wierd Kooistra,^a^ Monique E. Lodewijk,^a^ Malou Zuidscherwoude,^b^ Robert A. Pol,^f,g^ TransplantLines investigators,^g^ Edward. R. Smith,^h,i,j^ Guido Krenning,^k^ Jeroen H.F. de Baaij,^b^ Jan-Luuk Hillebrands,^a,l,**^ Joost G.J. Hoenderop,^b,l,*^

^a^Department of Pathology and Medical Biology, University of Groningen, University Medical Center Groningen, Hanzeplein 1, 9713 GZ, Groningen, The Netherlands

^b^Department of Medical Biosciences, Radboud university medical center, Nijmegen, The Netherlands

^c^Normandie Univ, UNICAEN, US EMerode, Plateform Proteogen, 14000 Caen, France

^d^Université de Caen Normandie, CNRS, Normandie Université, ISTCT UMR6030, GIP CYCERON, F-14000 Caen, France

^e^Department of Biomedical Sciences of Cells & Systems, University of Groningen, University Medical Center of Groningen, Antonius Deusinglaan 1, 9713 AV, Groningen, The Netherlands

^f^Department of Surgery, University of Groningen, University Medical Center Groningen, Hanzeplein 1, 9713 GZ Groningen, The Netherlands

^g^On behalf of the Transplantlines investigators

^h^Department of Nephrology, Royal Melbourne Hospital, Parkville, Victoria, Australia

^i^Department of Medicine, University of Melbourne, Parkville, Victoria, Australia

^j^Current address: SEHA Kidney Care, Abu Dhabi Health Services (SEHA), Abdu Dhabi, United Arab Emirates

^k^Department of Clinical Pharmacy and Pharmacology, University of Groningen, University Medical Center of Groningen, Hanzeplein 1, 9713 GZ, Groningen, The Netherlands

^l^Joint last authors

**CORRESPONDING AUTHORS**

^*^Prof. dr. Joost G.J. Hoenderop, Department of Medical Biosciences, Radboud university medical center, P.O. Box 9101, 6500HB, Nijmegen, The Netherlands. Phone: (+31) 24 3610574, E-mail: [joost.hoenderop@radboudumc.nl](mailto:joost.hoenderop@radboudumc.nl), ORCID: 0000-0002-1816-8544

^**^Prof. dr. Jan-Luuk Hillebrands, Department of Pathology and Medical Biology (HPC: EA10), University of Groningen, University Medical Center Groningen, Hanzeplein 1, 9713 GZ, Groningen, The Netherlands. Phone (+31) 06 25651329, E-mail: [j.l.hillebrands@umcg.nl](mailto:j.l.hillebrands@umcg.nl), ORCID: 0000-0003-3135-3274

**SUPPLEMENTARY FIGURES**


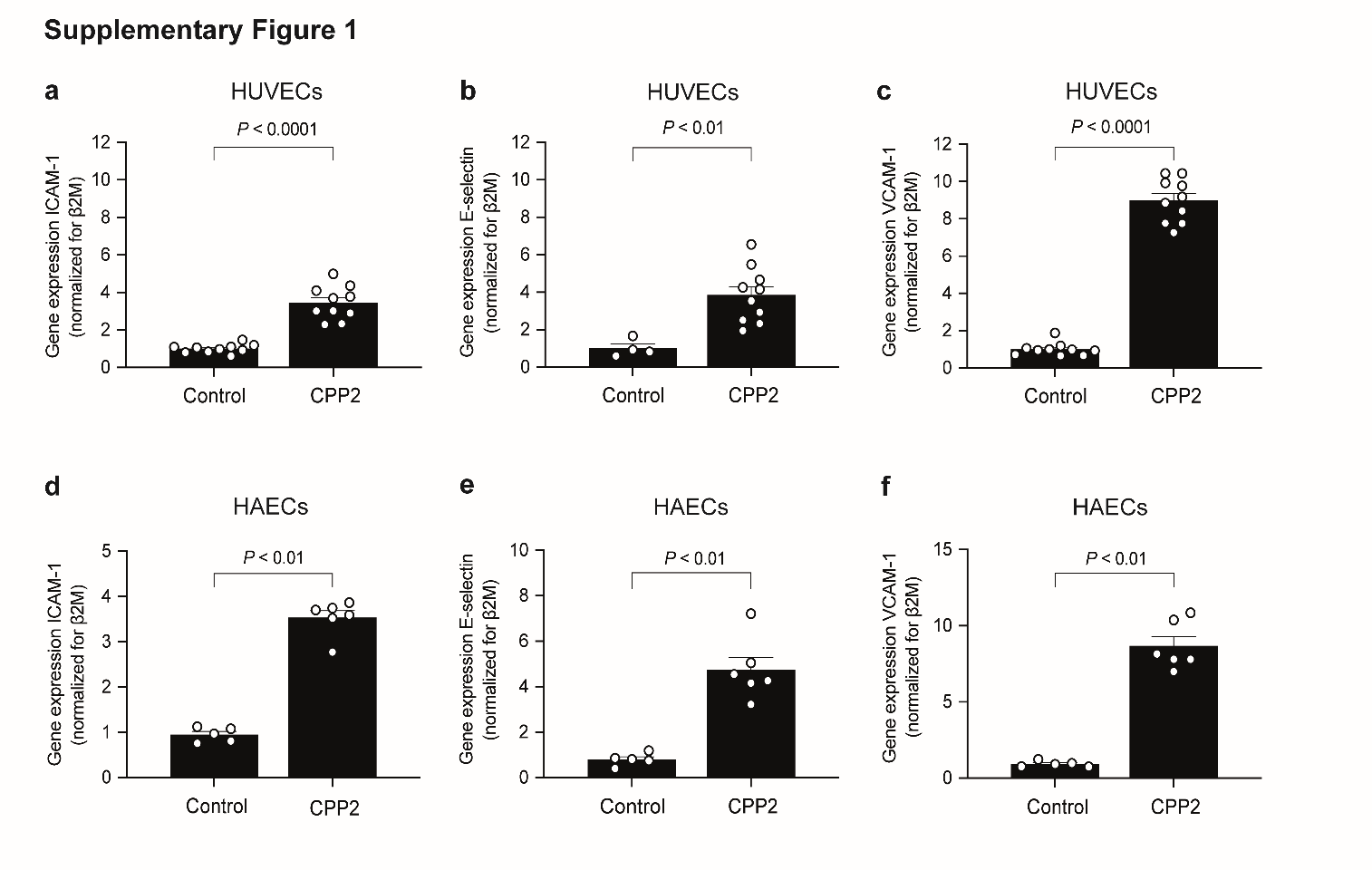


**Supplementary Figure S1. Validation of EC-activation marker mRNA expression in HUVECs and HAECs exposed to CPP2 (25 µg/ml) or control for 24h.** **(a)** ICAM-1 mRNA expression, **(b)** E-selectin mRNA expression, **(c)** VCAM-1 mRNA expression in HUVECs. **(d)** ICAM-1 mRNA expression, **(e)** E-selectin mRNA expression, **(f)** VCAM-1 mRNA expression in HAECs. Graphs show mean ± SEM and individual data points of two to four individual experiments (each consisting of 3 biological replicates). Missing data points reflect samples in which respective gene expression was below detection level. Mann-Whitney U test, *P*<0.05 considered statistically significant.

**
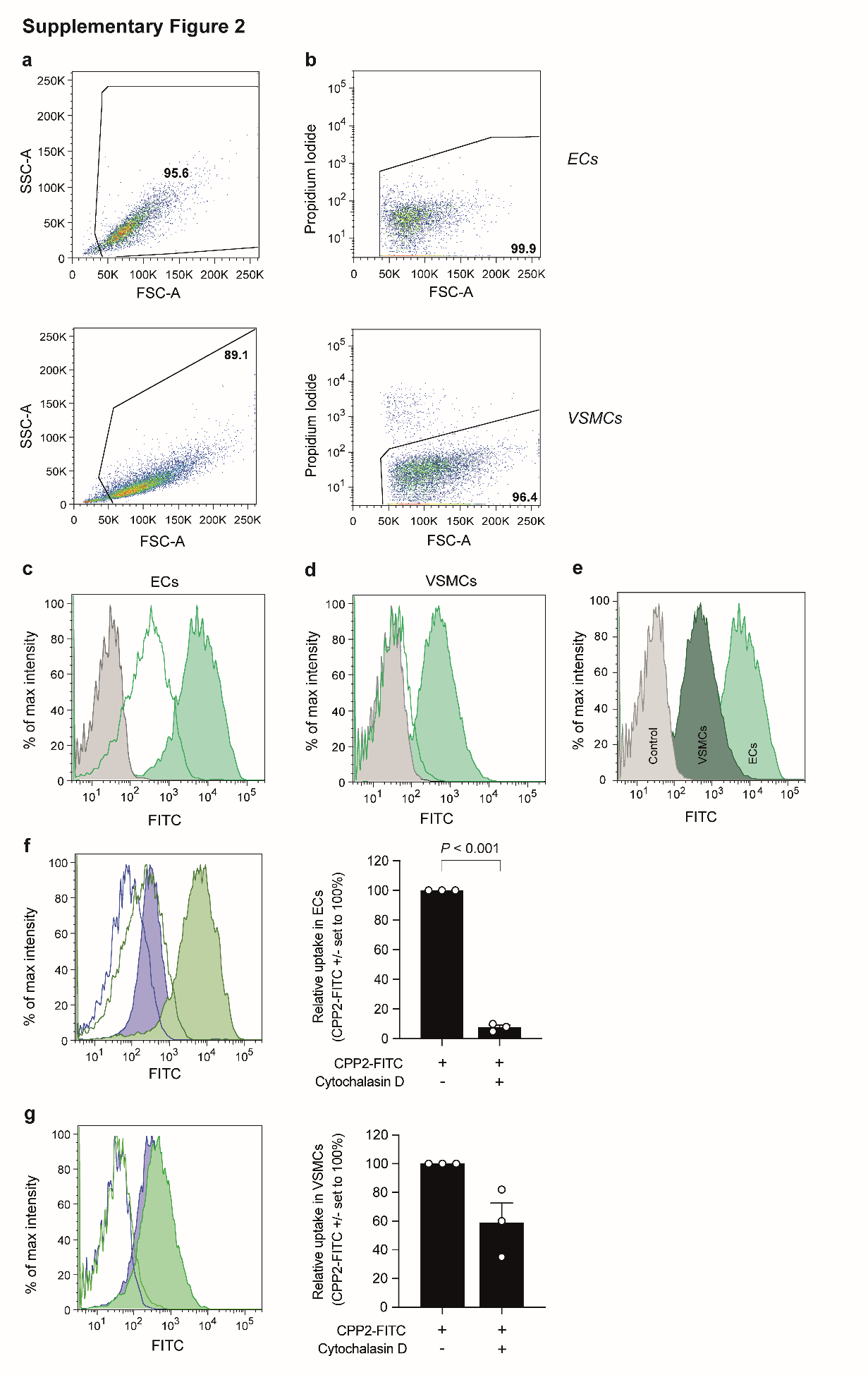

Supplementary Figure S2. FACS gating strategy and CPP cell uptake**. **(a)** Gating strategies with the forward scatter (FSC) plotted against the side scatter (SSC) to exclude debris in the sample. **(b)** Forward scatter plotted against the propidium iodide signal was used to exclude death cells in the analysis. Top graphs reflect the gating strategy for the ECs, while lower graphs for the VSMCs. Flow cytometric analysis quantified the uptake of FITC-labelled CPP2 (green) by ECs **(c)** and VSMCs **(d)** versus unstimulated ECs or VSMCs (no FITC-CPP2 added) (gray). Lines indicate incubation at 4°C (extracellular binding), filled histograms indicate incubation at 37°C (endocytosis + extracellular binding). **(e)** Comparison in fluorescent signal in ECs (light green) versus VSMCs (dark green) and unstimulated VSMCs (gray), showing more endocytosis of CPP2 by ECs. **(f)** 1 µM cytochalasin D (blue) suppresses endocytosis in ECs, but not in **(g)** VSMCs after CPP2 stimulation (green). Lines indicate incubation at 4°C (extracellular binding), filled histograms indicate incubation at 37°C (endocytosis + extracellular binding). Data is presented as mean ± SEM, each data point representing the mean of an individual experiment with three biological replicates. One sample t-test against a hypothetical value of 100%, *P*<0.05 considered statistically significant.

**
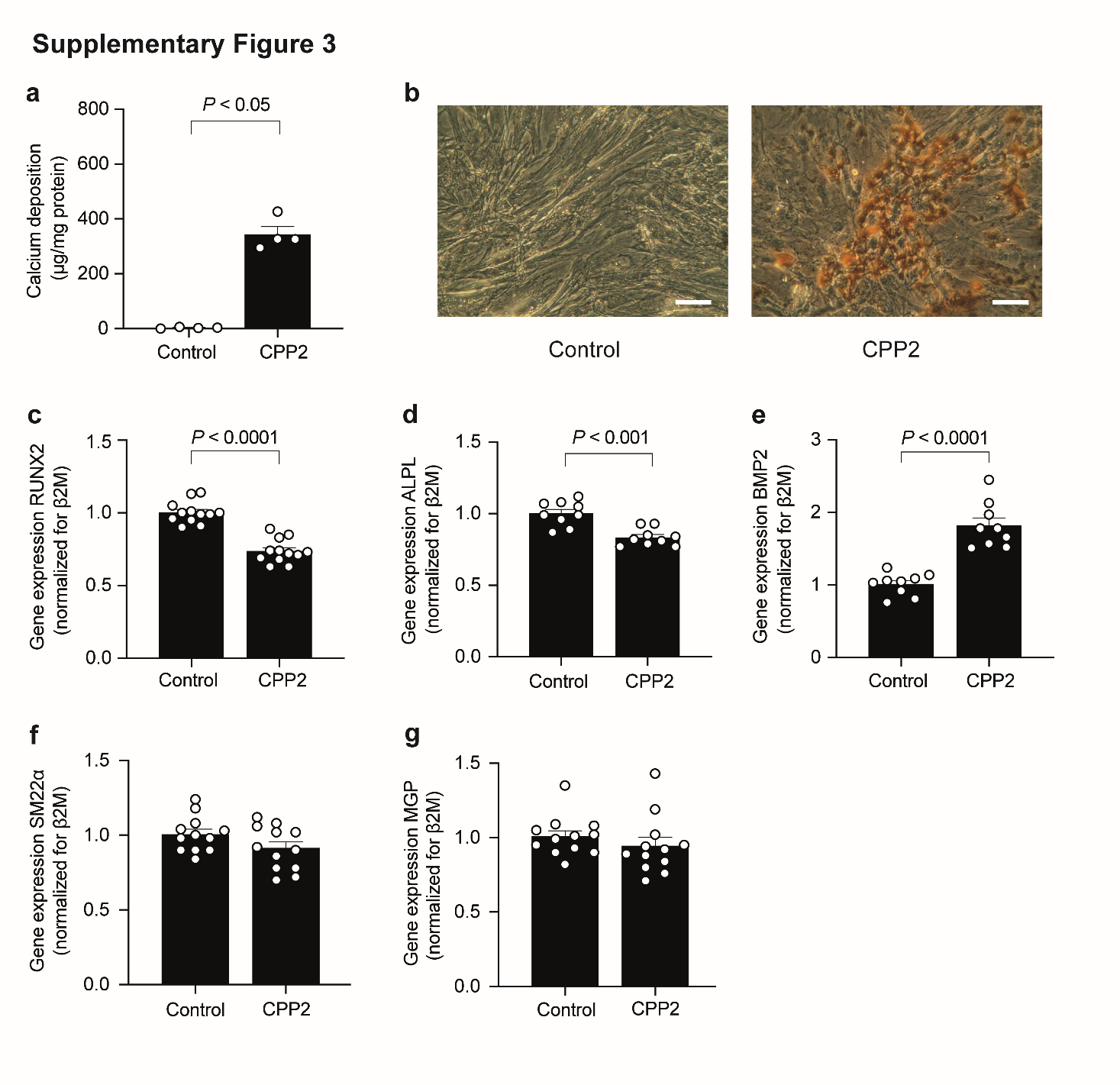
**

**Supplementary Figure S3. Calcium deposition and gene expression of VSMC dedifferentiation markers upon CPP2 stimulation. (a)** Calcium deposition measurements and **(b)** representative Alizarin red staining of VSMCs without (Control) and with CPP2 treatment (25 µg/ml). Scale bars represent 100 μm. Data in (**a**) is presented as mean ± SEM of n=4 individual experiments (each consisting of 3 biological replicates). Gene expression data of **(c)** Runt-related transcription factor 2 (RUNX2), **(d)** alkaline phosphatase (ALPL), **(e)** bone morphogenetic protein 2 (BMP2), **(f)** smooth muscle protein 22-alpha (transgelin, SM22⍺), and **(g)** matrix Gla protein (MGP) in VSMCs stimulated with or without CPP2. Gene expression data (**c-g**) derived from n=3-4 individual experiments (each consisting of 3 biological replicates). Mann-Whitney U test, *P*<0.05 considered statistically significant.

**
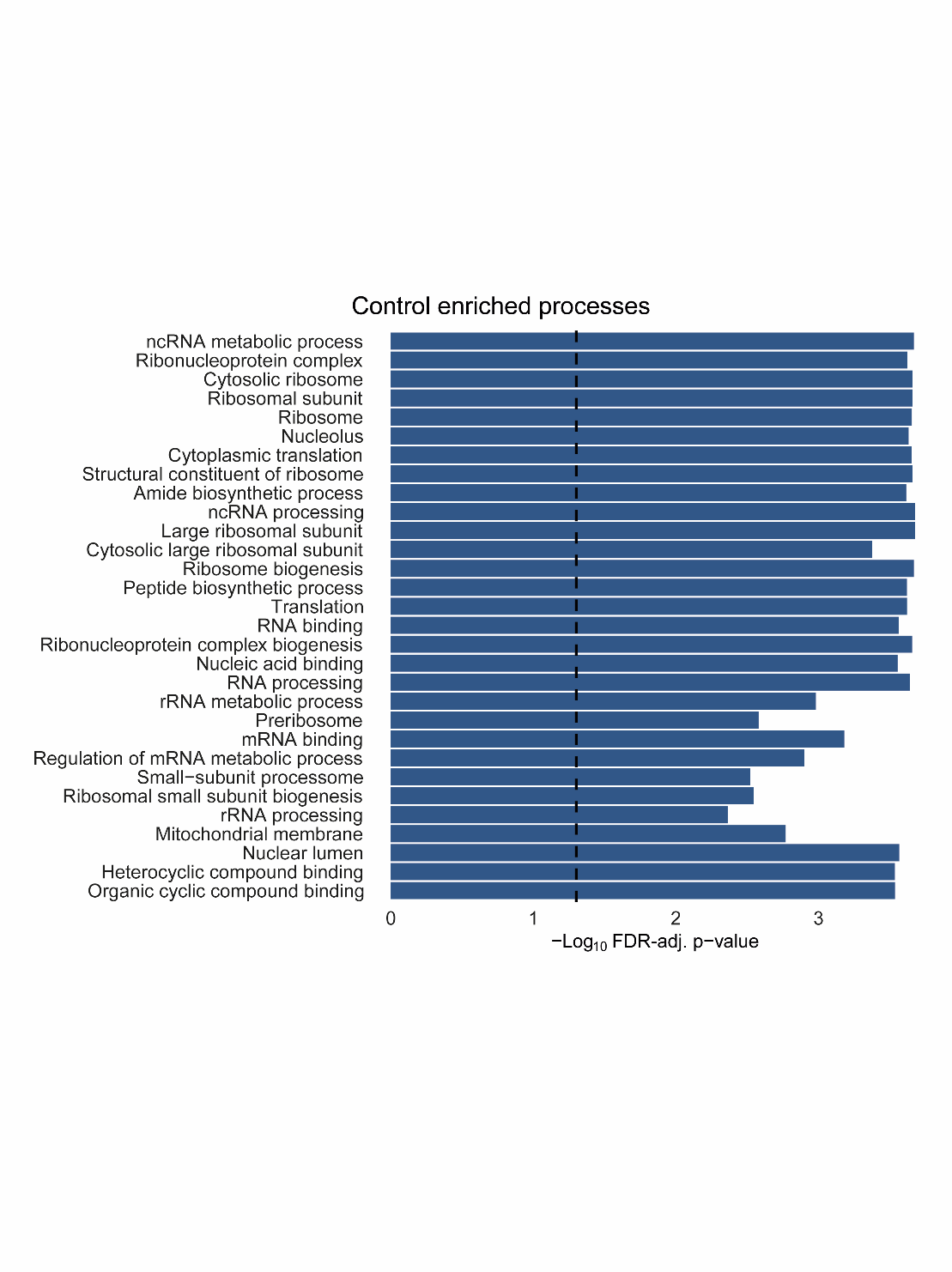
**

**Supplementary Figure S4. Gene set enrichment analysis (GSEA) of Gene Ontology (GO) terms using CluserProfiler including the differentially expressed proteins that were increased in the control (no CPP2) secretome.** Displayed are the top-30 enriched processes based on the control (no CPP2) secretome.

**SUPPLEMENTARY TABLES**

**Supplementary Table 1 –** Primer sequences

| **Gene** | **Forward sequence (5’ – 3’)** | **Reverse sequence (5’ – 3’)** |
| --- | --- | --- |
| ICAM-1 | CCTTCCTCACCGTGTACTGG | AGCGTAGGGTAAGGTTCTTGC |
| VCAM-1 | TGGACATAAGAAACTGGAAAAGG | CCACTCATCTCGATTTCTGGA |
| E-selectin | ACCAGCCCAGGTTGAATG | GGTTGGACAAGGCTGTGC |
| β2M | TTCTGGCCTGGAGGCTATC | TCAGGAAATTTGACTTTCCATTC |
| BMP2 | GGAACGGACATTCGGTCCTT | CACCATGGTCGACCTTTAGGA |
| MGP | CCTTCATATCCCCTCAGCAG | GCTTCCCTATTGAGCTCGTG |
| SM22α | GAAACCACCGGGGTGAGAG | GTCTGGGGAAAGCTCCTTGG |
| RUNX2 | TTACTGTCATGGCGGGTAACG | AAACTCTTGCCTCGTCCACTC |
| ALPL | CTATCCTGGCTCCGTGCTC | TTAACTGATGTTCCAATCCTGCG |

**Supplementary Table 2:** Differentially expressed genes in VSMC cultured in the presence of HUVEC-derived conditioned medium. HUVECs were cultured with (+ CPP2) or without (- CPP2) 25 µg/ml CPP2 for 24h.

| **Gene Name** | **Description** | **Fold change**  **(+ CPP2 *vs.* - CPP2)** | ***P*-value** |
| --- | --- | --- | --- |
| PCSK9 | proprotein convertase subtilisin/kexin type 9 | 2.70 | 0.03 |
| ACE | angiotensin I converting enzyme | 2.66 | 0.02 |
| IL33 | interleukin 33 | 2.19 | 0.002 |
| PTGS2 | prostaglandin-endoperoxide synthase 2 | 1.99 | 0.007 |
| CX3CL1 | chemokine (C-X3-C motif) ligand 1 (fractalkine) | 1.75 | 0.04 |
| ME1 | malic enzyme 1, NADP(+)-dependent, cytosolic | 1.33 | 0.03 |
| VEGFB | vascular endothelial growth factor B | 1.32 | 0.02 |
| SCD | stearoyl-CoA desaturase (delta-9-desaturase) | 1.29 | 0.004 |
| LRP4 | low density lipoprotein receptor-related protein 4 | -1.29 | 0.04 |
| MAPK11 | mitogen-activated protein kinase 11 | -1.30 | 0.02 |
